# Persistent Near-Linear Relationship Between Global Stress and Mean Atomic Bond Strain in Metallic Glasses Despite Significant Local Nonaffine Displacements

**DOI:** 10.3390/ma19102176

**Published:** 2026-05-21

**Authors:** Tittaya Thaiyanurak, Donghua Xu

**Affiliations:** 1 Materials Science Program, Oregon State University, Corvallis, OR 97331, USA; 2 School of Mechanical, Industrial and Manufacturing Engineering, Oregon State University, Corvallis, OR 97331, USA

**Keywords:** metallic glass, stress, strain, atomic bond, mechanical properties, deformation, elasticity, plasticity, shear transformation, shear band

## Abstract

Mean atomic bond strain (MABS), based on the globally averaged bond length, has recently emerged as a new strain metric that retains clear physical meaning even as severe atomic neighborhood reconstruction occurs. It has been shown to exhibit a nearly perfect linear relationship with global stress throughout the elastic and plastic deformation in single-crystal face-centered cubic (FCC) metals, contradicting conventional expectations based on nonlinear dislocation activity. Whether this near-linear relationship holds in other materials stands out as an important and intriguing question. In this study, we examine the MABS–stress relationship in representative unary, binary, and ternary metallic glasses (MGs), where neither a crystal structure nor dislocations are present. Large-scale molecular dynamics simulations of uniaxial tensile tests and statistical analysis of millions of atomic bonds are performed. Irrespective of their differing compositions, all the MGs exhibit a persistent near-linear relationship between total MABS (all bonds included) and global stress up to fracture, even in the presence of significant local nonaffine displacements (shear transformation zones and shear bands), with the Pearson correlation coefficient consistently exceeding 0.99. Unlike the nonaffine displacements, the spatial distribution of individual atomic bond strain does not localize under the uniaxial loading. In the MGs containing more than one element, MABS computed for a single bond type may not correlate as linearly with global stress as total MABS. The results demonstrate that the persistent near-linear total MABS–stress relationship over the entire deformation process, recently discovered in single-crystal FCC metals, also applies to MGs despite their vastly different atomic structures. This strengthens the candidacy of total MABS as a universal stress descriptor across materials classes and deformation regimes. With further development and implementation in atomistic simulations and constitutive modeling, the MABS concept has the potential to reshape our understanding of materials mechanics and generate new insights into the design of stronger, tougher, and more thermally and chemically stable materials.

## 1. Introduction

Atomic-scale strain is the fundamental origin of a material’s macroscopic mechanical behavior. It is typically evaluated for each atom in the form of the Green–Lagrange (G-L) tensor based on the local deformation gradient, following the formalism of continuum mechanics [1,2,3,4]. For each atom *i*, the local deformation gradient $F_{i}$ is obtained through an affine fit that minimizes this quantity, $D^{2}=\sum_{j}{\left\|r_{j}-r_{i}-F_{i}(r_{j}^{0}-r_{i}^{0})\right\|}^{2}$, where *j* refers to all neighbors of atom *i*, ***r*** is the atomic position in the current state, and ***r*^0^** is the atomic position in the reference state (e.g., at zero load). With the local deformation gradient known, the G-L strain tensor $E_{i}$ is calculated as $E_{i}=\frac{1}{2}\left(F_{i}^{T}F_{i}-I\right)$. Associated with this, the minimized $D^{2}$, i.e., $D_{min}^{2}$, represents the residual (or deviation) from the affine fit and is commonly referred to as the nonaffine squared displacements. The components of the G-L strain tensor, their derived quantities (e.g., von Mises shear strain), and $D_{min}^{2}$ are frequently used to identify and visualize localized deformation mechanisms, including dislocations in crystals, and shear transformation zones (STZs) and shear bands in metallic glasses (MGs) [2,3,5,6,7,8]. However, when the atomic neighborhood (i.e., neighbor identities) changes significantly due to sample yielding or plastic deformation, the assumptions underlying the deformation gradient, particularly the neighbor correspondence across states, break down. As a result, the quantitative values associated with the deformation gradient and G-L atomic strain are no longer physically meaningful, although they may still qualitatively highlight atoms experiencing higher strain.

Recently, we introduced a new strain metric, named atomic bond strain (ABS) or mean atomic bond strain (MABS), that does not rely on neighbor correspondence across states [9,10,11]. MABS calculates the average length of all existing bonds within the current state ($\langle l\rangle )$ and the reference state ($\langle l_{0}\rangle$), respectively, and then determines the mean bond strain as $\left(\langle l\rangle -\langle l_{0}\rangle \right)/\langle l_{0}\rangle$. Focusing only on the existing bonds in each state, MABS retains a clear physical meaning even when atomic neighborhoods are significantly reconstructed over time. The bond length to be averaged can be either the total length of a bond vector or the projected length along a specific direction, such as the loading direction in uniaxial tests. As shown in this paper, the ABS or MABS concept can be further adapted to examine a subset of bonds, or even individual bonds.

MABS was applied to the uniaxial deformation of single-crystal face-centered cubic (FCC) metals [11], where it showed a nearly perfect linear relationship with the global stress throughout the elastic and plastic regimes, even as dislocations were nucleated, moved, and annihilated. This contradicted the conventional understanding that plastic deformation is inherently nonlinear, and that both the nonlinearity and the plastic flow stress are dictated by complex dislocation dynamics in crystalline metals [12,13]. It was proposed that the apparent contradictions could be reconciled by viewing dislocation activities as temporary interruptions to bond stretching, with bond stretching directly controlling the plastic flow stress. Because atomic bonds exist in all materials, one intriguing question naturally arising from the previous study is whether the persistent near-linear relationship between global stress and MABS over the entire deformation range is unique to single-crystal FCC metals or more broadly present in other materials. This is a critical question, as it directly relates to the potential of MABS to bridge our understanding of deformation, particularly plasticity and stress, across material classes, and to serve as a simple, physics-based stress descriptor for more efficient constitutive modeling.

In this paper, we report our investigation of the MABS–stress relationship in MGs, represented by unary Ta, binary Ni_62_Nb_38_, and ternary Zr_47_Cu_46_Al_7_ (at.%). MGs possess vastly different atomic structures from single-crystal FCC metals, lacking periodic crystalline order and dislocations [14,15,16,17,18,19,20,21]. Their plastic deformation is accommodated by localized STZs (prior to yielding) and shear bands (post-yielding), as opposed to dislocations in crystalline materials [14,15,16,17,18,19,20,21,22,23,24,25,26,27]. Therefore, MGs provide a strong test of the broader applicability of the persistent near-linear relationship between MABS and global stress. The three representative MGs were selected for their good glass-forming ability, distinct compositions, varying compositional complexity, and the availability of quantum-mechanically based EAM (embedded atom method) potentials. In addition to the total MABS computed for all bonds, we also examine MABS for a single bond type (e.g., Zr-Zr) in the multicomponent MGs, and ABS for individual bonds using an adapted definition.

## 2. Simulation Methods

**Figure 1:**
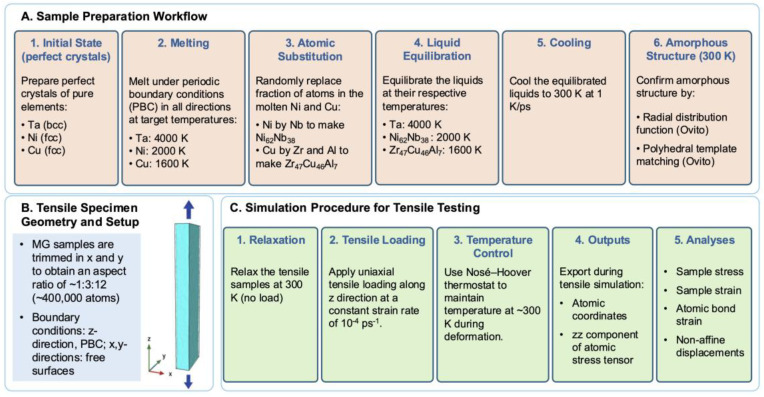
Schematics of the workflow and simulation parameters in this study.

Figure 1 presents schematics of the workflow and simulation parameters employed in this study. Large-scale molecular dynamics (MD) simulations were performed using the LAMMPS (Large-Scale Atomic/Molecular Massively Parallel Simulator) [28,29] code (version: 3 Mar 2020) with EAM potentials specifically developed for the Ta, Ni-Nb, and Zr-Cu–Al systems (https://sites.google.com/site/eampotentials for Ta and Zr-Cu-Al, and https://www.ctcms.nist.gov/potentials/ for Ni-Nb, accessed on 1 September 2025). To prepare MG samples of the target compositions, perfect crystals of pure Ta, Ni and Cu were first melted and equilibrated at 4000, 2000, and 1600 K, respectively, under periodic boundary conditions in all directions. Fractions of atoms in the molten Ni and Cu were then randomly replaced by alloying elements—Ni by Nb, and Cu by Zr and Al—according to the target compositions, after which the alloy liquids were further equilibrated at their respective temperatures. The equilibrated Ta, Ni_62_Nb_38_, and Zr_47_Cu_46_Al_7_ liquids were then cooled to 300 K at 1 K/ps. The resulting structures were all confirmed to be amorphous by radial distribution function (RDF) and polyhedral template matching using Ovito (Open Visualization Tool [30], version 2.9). The MG samples were subsequently trimmed in the x and y directions to produce specimens with an approximate aspect ratio of 1:3:12 and a total number of atoms on the order of 400,000. Periodic boundary conditions were removed in the x and y directions to allow free surface formation. All samples were relaxed at 300 K before being subjected to uniaxial tensile loading along the z direction at a constant strain rate of 10^−4^ ps^−1^. The Nosé–Hoover thermostat was used to maintain the temperature at ~300 K during deformation. Atomic coordinates and the zz component of atomic stress tensor were exported from the tensile simulations.

**Figure 2:**
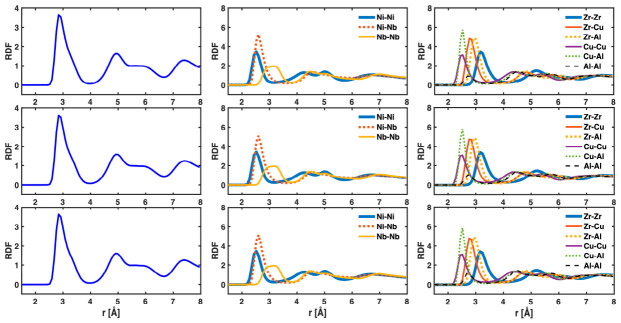
RDF or partial RDFs for Ta (**left column**), Ni_62_Nb_38_ (**center column**) and Zr_47_Cu_46_Al_7_ (**right column**) MGs at sample strains of 0 (**top row**) and 0.1 (**middle row**) and just before fracture (**bottom row**).

After the simulations, Ovito was used for visualization and analysis of atomic stress, global stress, G-L atomic strain, and ABS/MABS. In particular, global stress (z-direction) was computed as the volume-weighted average of the zz-atomic stress of all atoms, using the sample volume (as opposed to the cell volume) obtained via the Construct Surface Mesh modifier in Ovito. The G-L atomic strain and associated $D_{min}^{2}$ were computed using the Atomic Strain modifier, with the relaxed state prior to tensile loading as the reference. For bond strain analysis, bonds were identified using bond-type-specific cutoff distances (listed in Table 1), determined from the minimum between the first and second peaks in the RDF (or, partial RDFs for the binary and ternary MGs), which remained virtually unchanged during the tensile process—as shown in Figure 2. Note that these RDFs are non-directional and include contributions from all directions, rather than only the tensile direction. Non-directional RDFs are required for defining the bond cutoff distances in the full 3D space. Also note that the RDFs directly from LAMMPS or Ovito are based on the simulation cell volume. They have been corrected here with the sample volume. To perform statistical analysis on the multi-million bonds, a custom Python (version 3.5.2) script was used within Ovito which allowed efficient averaging of z-lengths over all bonds or a specific type of bonds. More details about the stress and strain analysis can be found in Refs. [9,10,11].

## 3. Results and Discussion

**Figure 3:**
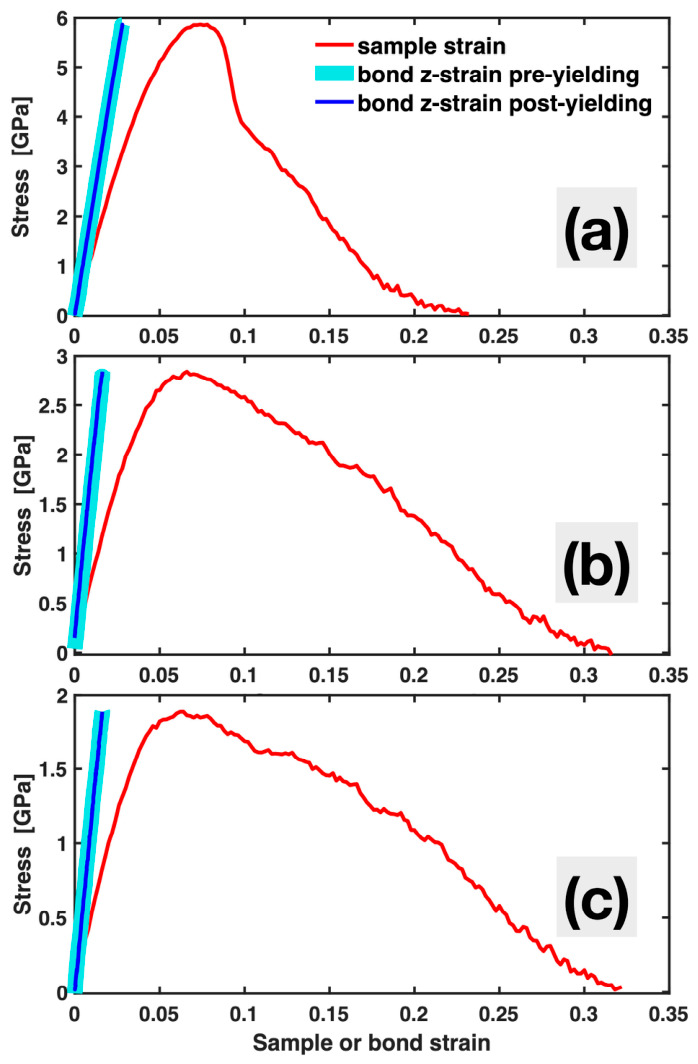
Global stress plotted against sample strain and bond z-strain (total MABS), the latter divided into pre-yielding and post-yielding segments, for Ta (**a**), Ni_62_Nb_38_ (**b**) and Zr_47_Cu_46_Al_7_ (**c**) MGs.

The conventional stress–strain curves, i.e., plots of global stress versus sample strain, both in the tensile (z) direction, are presented in Figure 3 as thin red lines for the three representative MGs. They are typical of MD-simulated tensile tests on MGs, each containing an initial ascending segment that is largely elastic (with a noticeable, gradual change in slope), followed by yielding and nonlinear post-yielding plastic deformation. In experimental tensile tests with bulk (millimeter- to centimeter-scale) MG samples, smaller sample strains than in MD are usually observed before fracture. However, in certain dedicated nanoscale tensile experiments, sample strains approaching the typical MD results have been reported. These differences in apparent sample strains are likely related to sample size effects, which are not the focus here. For the present study, the substantial sample strain in MD, particularly the post-yielding plastic strain, in fact provides a strong test of the stress–MABS relationship in MGs.

Also included in Figure 3 are the plots of the global stress versus total MABS (computed for all existing bonds with their projected z-lengths) for the three MGs. For clarity, each of these plots is divided into two segments: pre-yielding (thick cyan line) and post-yielding (thin blue line). It is evident that the relationship between global stress and the total MABS in all the MGs is highly linear, both before and after yielding, with a nearly constant slope representing an overall bond modulus that is unchanged by yielding or deformation. This persistent near-linear stress–total MABS relationship is the same as previously observed in single-crystal FCC metals, even though MGs have vastly different atomic structures and deformation mechanisms from single-crystal FCC metals. This suggests a fundamental role of the total MABS, or the overall bond stretching, in governing the global stress, which is not limited to a particular class of materials, deformation regime, or mechanism.

**Figure 4:**
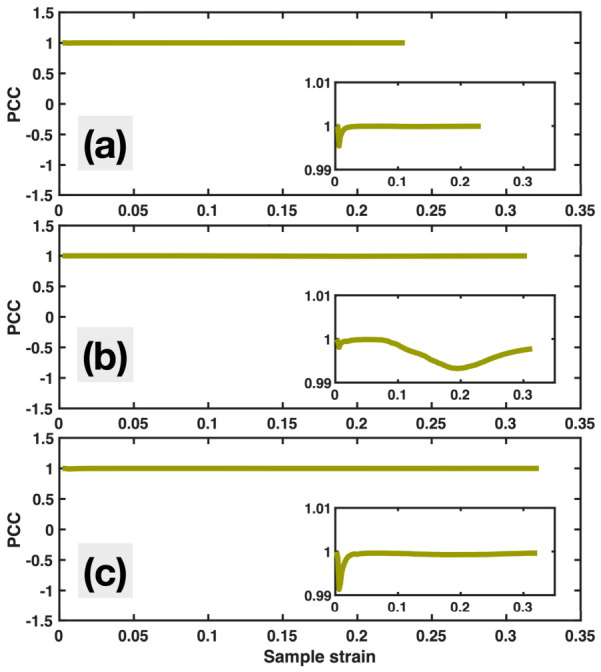
Cumulative Pearson correlation coefficient (PCC) for the total MABS–stress relationship, plotted against sample strain in Ta (**a**), Ni_62_Nb_38_ (**b**) and Zr_47_Cu_46_Al_7_ (**c**) MGs. The insets are zoomed in along the vertical axes to show how close the PCC values are to the ideal value of one.

To quantify the near-linear stress–total MABS relationship, we calculated their Pearson correlation coefficient (PCC), which is defined as $PCC=\frac{\sum_{i=1}^{n}\left(x_{i}-\bar{x}\right)\left(y_{i}-\bar{y}\right)}{\sqrt{\sum_{i=1}^{n}{\left(x_{i}-\bar{x}\right)}^{2}}\sqrt{\sum_{i=1}^{n}{\left(y_{i}-\bar{y}\right)}^{2}}}$ for any two data sets *x* and *y*. By definition, PCC can range from −1 to +1, with +1 indicating a perfect positive linear correlation. Figure 4 presents the cumulative PCC values, calculated on a rolling basis from the start of the tensile loading, plotted against sample strain over the entire deformation process. The main panels in the figure show that PCC remains close to one at all times for all three MGs. The insets further reveal that all PCC values exceed 0.99, suggesting an almost perfect linear correlation between the stress and total MABS.

Alongside the persistent near-linear stress–total MABS relationship, one additional feature exhibited by the MGs in Figure 3, not previously observed in single-crystal FCC metals, is that the pre-yielding segments of the conventional stress–sample strain curves deviate gradually from the stress–total MABS plots. In a single-crystal FCC metal, the material behavior is almost entirely elastic before yielding; therefore, the pre-yielding segment of the stress–sample strain curve coincides with the stress–MABS plot. In MGs, in contrast, local plastic deformation in the form of STZs (i.e., small groups of atoms undergoing cooperative shear) takes place well before yielding, and the STZ population increases with stress. This is the main reason for the gradual change in slope of the pre-yielding segments of the stress–sample strain curves and their deviation from the stress–MABS plots exhibited in Figure 3.

**Figure 5:**
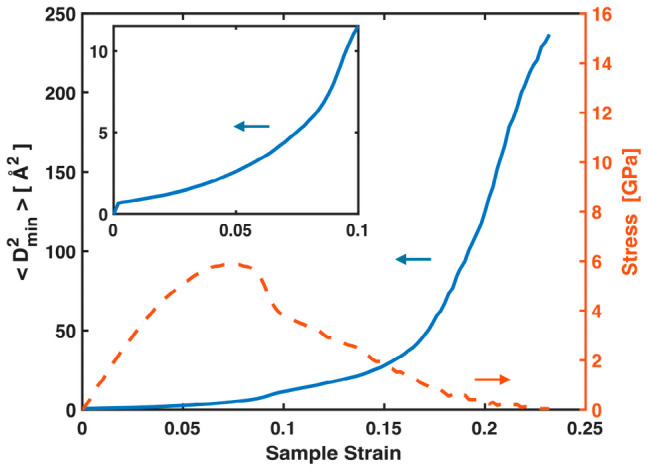
(Main panel) Averaged nonaffine square displacements $\langle D_{min}^{2}\rangle$ and global stress plotted against sample strain for the Ta MG. (Inset) Enlarged view of the initial segment of the $\langle D_{min}^{2}\rangle$ vs. sample strain plot.

STZs in MGs, as well as their aggregated form—shear bands that develop at yielding—manifest as local nonaffine displacements. These events and the atoms involved are typically identified using $D_{min}^{2}$. Figure 5 plots $\langle D_{min}^{2}\rangle$, the nonaffine squared displacements averaged over all atoms, versus sample strain, together with the stress–sample strain curve for the Ta MG. While its exact values in the plastic regime may not be reliable due to its association with the G-L strain tensor and deformation gradient discussed in the Introduction, the continuous increase of $\langle D_{min}^{2}\rangle$ with sample strain, even in the early pre-yielding stage, indicates a progressive accumulation of local nonaffine deformation. Near and after yielding, $\langle D_{min}^{2}\rangle$ increases more rapidly, reflecting intensified local nonaffine deformation through both STZ activity and shear banding. These nonaffine events play a significant role in the conventional stress–sample strain curves, contributing to their nonlinearity both before and after yielding. Nevertheless, throughout the entire deformation process, they have virtually no impact on the near-linear relationship between the total MABS and stress, as shown in Figure 3 and Figure 4. STZs and shear bands reconstruct local bonds and relax the full bond network, but the global stress at any time is governed by the total stretching of all bonds, pre-existing or newly formed.

**Figure 6:**
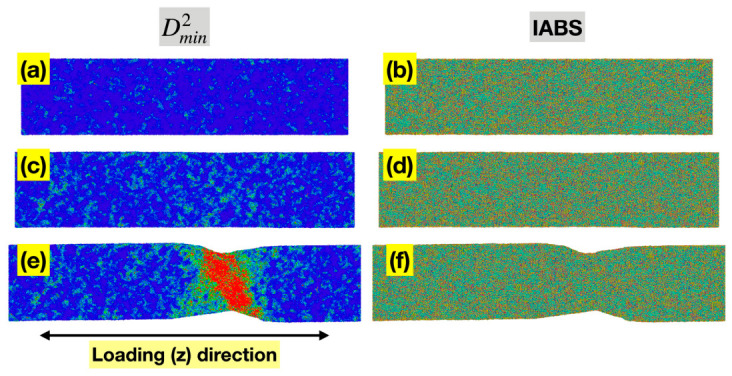
Spatial distributions of $D_{min}^{2}$ (**a**,**c**,**e**) and IABS (z-component) (**b**,**d**,**f**) for the Ta MG at sample strains of 0.04 (**top row**), 0.08 (**middle row**), and 0.12 (**bottom row**), representing the pre-yielding, near-yielding, and post-yielding regimes, respectively. In all panels, red denotes high values and blue denotes low values.

Figure 6a,c,e shows the spatial distribution of per-atom $D_{min}^{2}$ for the Ta MG at sample strains of 0.04, 0.08, and 0.12, representing the pre-yielding, near-yielding, and post-yielding regimes, respectively. In all three states, the higher $D_{min}^{2}$ values are clearly localized, either as small groups of atoms (STZs) or in a shear band. This is consistent with the current understanding of deformation mechanisms in MGs and with the nonlinearity in the conventional stress–sample strain curves in Figure 3.

Figure 6b,d,f presents the spatial distribution of ABS of individual bonds (IABS) for the same three states of the Ta MG. IABS here is defined as $\left(l_{i}-\langle l_{0}\rangle \right)/\langle l_{0}\rangle$ for each currently existing bond *i*, where $\langle l_{0}\rangle$ is the mean bond length of all bonds at zero load. Under their definitions, MABS equals the statistical average of IABS since $\langle \left(l_{i}-\langle l_{0}\rangle \right)/\langle l_{0}\rangle \rangle =\left(\langle l\rangle -\langle l_{0}\rangle \right)/\langle l_{0}\rangle$. Similarly to MABS, IABS can be evaluated either based on the full length of a bond vector, or the projected length in a certain direction. Here we are focusing on the loading (z) direction. Unlike $D_{min}^{2}$, the spatial distribution of IABS is rather uniform in all three states, without noticeable localization, implying that the redistribution of ABS across the entire bond network is highly efficient, even as significant localized nonaffine deformation occurs. This contributes to the persistent near-linear relationship between the global stress and total MABS observed in Figure 3.

**Figure 7:**
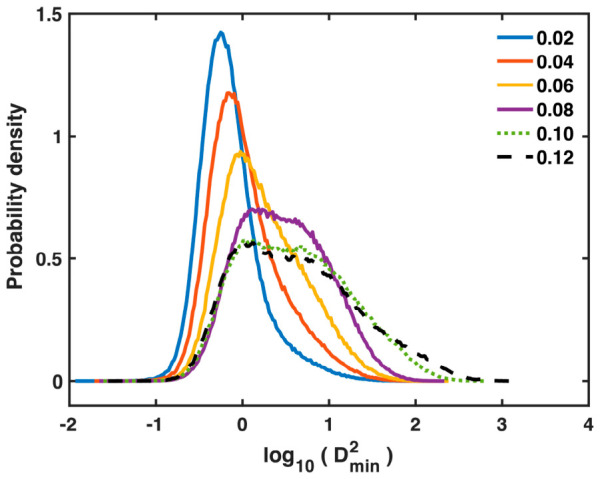
Probability density of ${log}_{10}\left(D_{min}^{2}\right)$ for all atoms in the Ta MG at increasing sample strains (as denoted in the legend). Note that yielding occurs around a sample strain of 0.08.

In addition to the mean and spatial distribution, we also examined the statistical (probabilistic) distributions of $D_{min}^{2}$ among all atoms and IABS among all bonds to better understand their evolution during deformation. Because a small fraction of atoms associated with localized STZs and shear bands possesses much larger and broader $D_{min}^{2}$ values than the majority of atoms, as shown in Figure 6, the statistical evolution of $D_{min}^{2}$ is best captured using the probability density of ${log}_{10}\left(D_{min}^{2}\right)$, which is plotted in Figure 7 for the Ta MG at six representative sample strains. In the early deformation stage, the probability density gradually shifts toward larger $D_{min}^{2}$ values as the sample strain increases from 0.02 to 0.04 and 0.06. Near yielding, at a sample strain of 0.08, a shoulder emerges on the right side of the probability density curve and significantly broadens the distribution peak, indicating an accelerated flow of probability into larger $D_{min}^{2}$ values. After yielding, the distribution continues to broaden toward larger $D_{min}^{2}$ values, extending beyond 1600 Å^2^ (${log}_{10}\left(D_{min}^{2}\right)=3.206)\;$at a sample strain of 0.12. All these features are consistent with the behavior of the mean $D_{min}^{2}$ shown in Figure 5.

**Figure 8:**
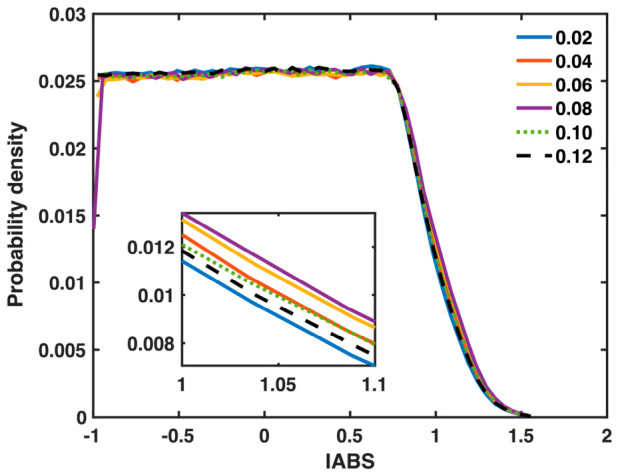
Probability density of IABS (z-component) for all bonds in the Ta MG at increasing sample strains (as denoted in the legend). The inset is an enlarged view of the curves in a high-value range. Note that yielding occurs around a sample strain of 0.08.

Figure 8 presents the probability density of IABS (z-component) for all bonds in the Ta MG at the same series of sample strains used in Figure 7. The main panel shows that the changes in the statistical distribution of IABS caused by deformation are rather subtle, consistent with the small maximal MABS value (0.028) shown in Figure 3a. The enlarged view shown in the inset reveals that the probability density at larger IABS values gradually increases in the early deformation stage (sample strain = 0.02, 0.04, and 0.06), reaches a maximum near yielding (sample strain = 0.08), and then decreases as the sample strain increases to 0.10 and 0.12. These features are consistent with the MABS behavior shown in Figure 3a.

**Figure 9:**
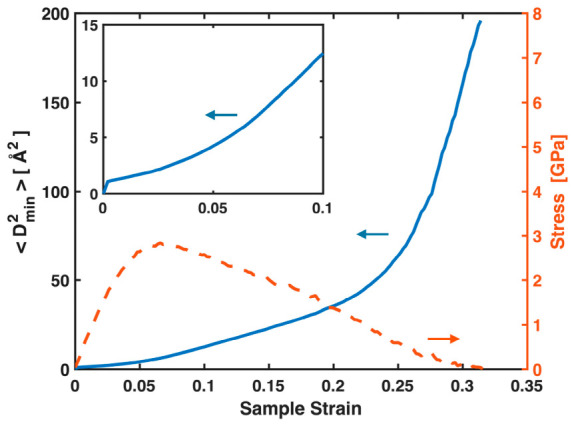
(Main panel) Averaged nonaffine square displacements $\langle D_{min}^{2}\rangle$ and global stress plotted against sample strain for the Ni_62_Nb_38_ MG. (Inset) Enlarged view of the initial segment of the $\langle D_{min}^{2}\rangle$ vs. sample strain plot.

**Figure 10:**
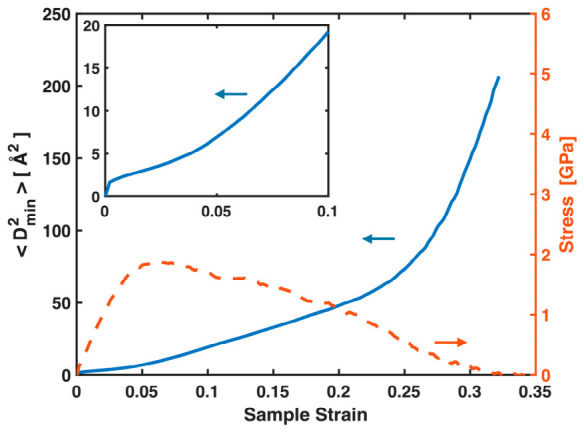
(Main panel) Averaged nonaffine square displacements $\langle D_{min}^{2}\rangle$ and global stress plotted against sample strain for the Zr_47_Cu_46_Al_7_ MG. (Inset) Enlarged view of the initial segment of the $\langle D_{min}^{2}\rangle$ vs. sample strain plot.

**Figure 11:**
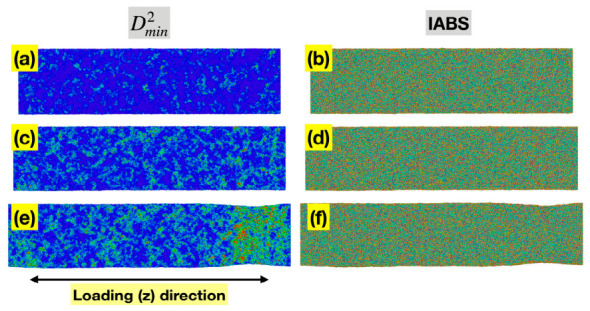
Spatial distributions of $D_{min}^{2}$ (**a**,**c**,**e**) and IABS (z-component) (**b**,**d**,**f**) for the Ni_62_Nb_38_ MG at sample strains of 0.04 (**top row**), 0.08 (**middle row**), and 0.12 (**bottom row**), representing the pre-yielding, near-yielding, and post-yielding regimes, respectively. In all panels, red denotes high values and blue denotes low values.

**Figure 12:**
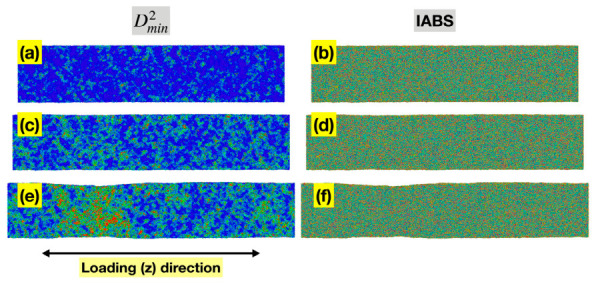
Spatial distributions of $D_{min}^{2}$ (**a**,**c**,**e**), and IABS (z-component) (**b**,**d**,**f**) for the Zr_47_Cu_46_Al_7_ MG at sample strains of 0.04 (**top row**), 0.08 (**middle row**), and 0.12 (**bottom row**), representing the pre-yielding, near-yielding, and post-yielding regimes, respectively. In all panels, red denotes high values and blue denotes low values.

The above discussions on the nonaffine squared displacements, IABS and their spatial and statistical distributions in Ta are also applicable to the Ni_62_Nb_38_ and Zr_47_Cu_46_Al_7_ MGs, and representative results are provided in Figure 9, Figure 10, Figure 11 and Figure 12 for reference.

**Figure 13:**
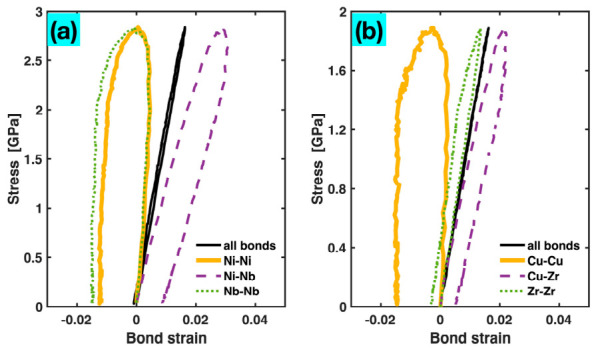
Stress plotted vs. total MABS (all bonds) and bond-type-specific MABS for the Ni_62_Nb_38_ (**a**) and Zr_47_Cu_46_Al_7_ (**b**) MGs. For clarity, the bond-type-specific MABS curves for only Cu-Cu, Cu-Zr, and Zr-Zr bonds are shown for the ternary MG.

In the unary Ta MG, all bonds are of the same type. However, this is not the case for the binary and ternary MGs. For example, in the ternary Zr_47_Cu_46_Al_7_, there are six distinct bond types, including like bonds (e.g., Zr-Zr) and unlike bonds (e.g., Cu-Zr). It is of interest to examine how the overall stretching of bonds of a specific type relates to the stress. Therefore, we evaluated bond-type-specific MABS, employing the same mathematical formalism as for the total MABS, i.e., $\left(\langle l\rangle -\langle l_{0}\rangle \right)/\langle l_{0}\rangle$, but restricting $\langle l\rangle$ and $\langle l_{0}\rangle$ calculations to a single bond type only. Figure 13 displays the plots of the global stress versus specific MABS computed for the Ni-Ni, Ni-Nb, and Nb-Nb bonds in the Ni_62_Nb_38_ MG and the Cu-Cu, Cu-Zr, and Zr-Zr bonds in the Zr_47_Cu_46_Al_7_ MG, along with the corresponding stress–total MABS (all bonds included) plots. All these plots start from the origin, rise with stress during the pre-yielding stage, reach their respective maxima near yielding, and then decline as the stress decreases after yielding. For the stress–total MABS plots, the pre-yielding and post-yielding segments nearly overlap and are both close to a straight line, indicating an almost perfect linear correlation between stress and total MABS, as already shown in Figure 3 and Figure 4. For the stress vs. bond-type-specific MABS plots, however, a noticeable separation is observed between the pre-yielding and post-yielding segments, and both segments exhibit greater nonlinearity than the stress–total MABS plots. The exact shape of the bond-type-specific MABS plot differs across bond types. These are due to the varying bond strengths and behaviors under load (e.g., stretching versus reconstruction) among the different bond types, as reported in our previous publication [9]. To briefly recap, the stronger bonds—particularly unlike atomic pairs with large negative heats of mixing, such as Ni-Nb in Ni_62_Nb_38_ and Cu-Zr in Zr_47_Cu_46_Al_7_—are stretched more than the weaker bonds, resulting in more positive bond-type-specific MABS throughout the tensile deformation process, even after sample fracture. In contrast, weaker bonds such as Ni-Ni and Cu-Cu yield (bond yielding can be separately defined for each bond type—see Ref. [9]) and reconstruct more easily, leading to overall less stretched or even shortened bond z-lengths, i.e., less positive or even negative values of bond-type-specific MABS. It is useful to reiterate that, despite the diverging behaviors of different bond types, the global stress remains highly linear with the total MABS, highlighting the collective role of all bonds in governing the overall stress.

## 4. Conclusions

By performing large-scale MD tensile simulations and bond statistical analysis, we have examined our recently introduced strain metric, the atomic bond strain, and its relationship with global stress in unary (Ta), binary (Ni_62_Nb_38_) and ternary (Zr_47_Cu_46_Al_7_) metallic glasses (MGs). Regardless of their compositions, all the MGs exhibit a persistent near-linear relationship between the total mean atomic bond strain (with all bonds included) and global stress throughout the deformation process, even as atoms undergo significant local nonaffine displacements (shear transformation zones and shear bands). The Pearson correlation coefficient between the two is consistently above 0.99. This suggests that the global stress is predominantly determined by the overall stretching of the entire bond network at all times. Unlike the nonaffine displacements, the atomic bond strain for individual bonds does not localize, indicating efficient redistribution of bond stretching across the bond network. In the MGs containing more than one element, mean atomic bond strain computed for any specific bond type is less linearly related to the global stress than the total mean atomic bond strain due to diverse characteristics of different bond types. The results demonstrate that the near-linear global stress–total mean atomic bond strain relationship over the full deformation range, recently discovered in single-crystal FCC metals, also holds in MGs, despite their vastly different atomic structures and deformation mechanisms. The study also provides deeper insights into individual atomic bond strain and bond-type-specific mean atomic bond strain, along with examples of their analysis, which can be extended to other amorphous or crystalline material systems.

While significantly corroborated by the results reported here, the universality of the near-linear relationship between the global stress and the total mean atomic bond strain remains to be tested in more material systems (e.g., polycrystalline and quasicrystalline materials) through molecular dynamics simulations. Experimentally, direction-resolved radial distribution functions obtained from synchrotron measurements can potentially be used to validate this relationship in the elastic and uniform plastic regimes, although a more reliable method for extracting projected mean bond strain than simple peak/valley shifting has yet to be established.

As discussed in our previous publication [11], the atomic bond strain concept, upon further development, has the potential to impact multiple areas of research. It could provide a unified framework for the fundamental accounts of stress and strain, linking elasticity with plasticity, atomic-scale with continuum mechanics, and crystalline with amorphous materials, which are traditionally treated as separate domains. Practically, through broader implementation in future atomistic simulations and constitutive modeling, it may generate new insights into the design of stronger, tougher, and more thermally and chemically stable materials from the perspective of bond–strain behavior.

## Figures and Tables

**Table 1:** Cutoff distances for different types of bonds in the representative MGs.

MG	Ta	Ni_62_Nb_38_			Zr_47_Cu_46_Al_7_					
Bond type	Ta-Ta	Ni-Ni	Ni-Nb	Nb-Nb	Zr-Zr	Zr-Cu	Zr-Al	Cu-Cu	Cu-Al	Al-Al
Cutoff distance (Å)	4.0	3.1	3.6	3.8	4.1	3.7	3.9	3.2	3.3	3.4

## Data Availability

The data that support the findings of this study are available from the corresponding author upon reasonable request.
